# The role of the cantilever in Kelvin probe force microscopy measurements

**DOI:** 10.3762/bjnano.2.29

**Published:** 2011-05-18

**Authors:** George Elias, Thilo Glatzel, Ernst Meyer, Alex Schwarzman, Amir Boag, Yossi Rosenwaks

**Affiliations:** 1 School of Electrical Engineering, Faculty of Engineering, Tel-Aviv University, Ramat-Aviv 69978, Israel; 2 Department of Physics, University of Basel, Basel 4056, Switzerland

**Keywords:** boundary elements method, cantilever, convolution, Kelvin probe force microscopy, point spread function

## Abstract

The role of the cantilever in quantitative Kelvin probe force microscopy (KPFM) is rigorously analyzed. We use the boundary element method to calculate the point spread function of the measuring probe: Tip and cantilever. The calculations show that the cantilever has a very strong effect on the absolute value of the measured contact potential difference even under ultra-high vacuum conditions, and we demonstrate a good agreement between our model and KPFM measurements in ultra-high vacuum of NaCl monolayers grown on Cu(111). The effect of the oscillating cantilever shape on the KPFM resolution and sensitivity has been calculated and found to be relatively small.

## Introduction

The effect of the measuring probe in electrostatic force based microscopies, such as Kelvin probe force microscopy (KPFM) [1], is very large because the measured forces are long range. This effect has been studied and analyzed by several groups [2–9], who invariably focused on the contribution of the tip while neglecting the effect of the cantilever or took it into account using various approximations. Hochwitz et al. [10] and Belaidi et al. [11] estimated the entire cantilever contribution to the overall electrostatic force as a function of the probe–sample distance and cantilever–sample angle. They concluded that the cantilever may impose a limitation on the maximal probe–sample distance that can be used to obtain high lateral resolution. Colchero et al. [12] calculated the influence of the cantilever on the KPFM resolution, and several groups [13–15] derived analytic expressions for the cantilever electrostatic force. To the best of our knowledge, despite the above studies, the accurate role of the cantilever in general, and in high resolution ultra-high vacuum (UHV) KPFM measurements in particular, has not been reported. In this work we use the boundary element method (BEM) [7] to calculate the point spread function (PSF) of the measuring probe: Tip and cantilever. The probe PSF analysis shows that the cantilever has a very strong effect on the absolute value of the measured contact potential difference (CPD) even under UHV conditions, and we demonstrate a good agreement between our model and KPFM measurements.

## Experimental

### Electrostatic model

In order to calculate the full probe configuration, we extended our previous model [7] to solve the entire probe–surface electrostatic system, including the cantilever. The model assumes a conducting probe and a sample that is represented by an infinitely thin dipole layer on top of an earthed plane; variations in the dipole density account for the inhomogeneous sample surface potential. Both the probe and the sample were divided into boundary elements in order to calculate their surface charge density. Unlike our previous work [7], where the probe was divided into conical and spherical elements, here we used commercial software (MSC/Patran®) in order to perform fast automatic meshing of an arbitrary probe geometry, including the cantilever as required in this work.

The probe charge density was used as the unknown quantity to be determined in order to calculate subsequently the PSF. We use the following notations: (a) A matrix **G** which is a discrete representation of the Green’s function between two probe boundary elements; (b) a matrix **D** which represents the discretized influence of the dipole layer (representing the sample) on each probe mesh element; (c) a diagonal matrix **B** with diagonal elements equal to the *z* components of the normal area vectors of the probe boundary elements divided by 2ε_o_, and (d) the vector 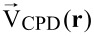, which is a discrete representation of the surface potential, corresponding to a probe centered at **r** = (*x,y,z*). Matrices **G**, **D**, **B** and vector 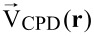 were previously defined in [7] and are explained again in the Appendix section.

The probe–sample system was solved by dividing the mutual interactions into homogeneous and inhomogeneous parts. The homogeneous part represents a system with a probe above an infinite earthed plane, while the inhomogeneous part accounts for the contribution of the sample surface potential to the electrostatic force acting on the probe; the total potential is a sum of the two parts. In addition, we define 
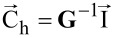
, and **C**_inh_ = **G**^−1^ **D** where **G**^−1^ is the inverse of **G**, and 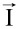 is a vector with all elements equal to 1. The vector 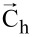 represents the capacitance density (capacitance per unit area) between two probe elements and the matrix **C**_inh_ represents the mutual capacitance density between every pair of surface and probe elements. By inserting the charge density distribution into the Maxwell stress tensor, replacing the probe potential with *V*_dc_(**r**) + *V*_ac_ sin(ω*t*), and extracting the force, we obtained the following expression for the electrostatic force acting on the entire probe in the *z* direction at frequency ω:

[1]
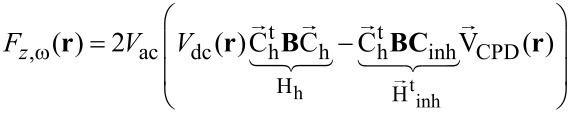


where H_h_ is the coefficient of the homogeneous force component, and 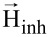 scales the relative contribution of each sample element to the inhomogeneous force; the superscript t denotes the transpose vector. The distinction between the homogeneous and inhomogeneous parts of the force is not merely mathematical; while the homogeneous force depends on the applied voltage, *V*_dc_, the inhomogeneous force is proportional to a weighted average of the sample potential. These weights are due to the contributions from areas at different distances from the probe, and therefore will determine the KPFM spatial resolution.

Equation 1 calculates the force for a specific probe–sample distance. In practice, almost all UHV KPFM measurements use the single pass method. In this method, the cantilever oscillates at its first resonance frequency in order to measure the surface topography, while the oscillations due to the electrostatic force (in amplitude modulated AM-KPFM at the second resonance or in frequency modulated FM-KPFM at several hundred Hz [16]) are nullified by adjusting *V*_dc_(**r**). The first resonance oscillations have a strong effect on the measured CPD, especially at probe–sample distances smaller than 10 nm, where the electrostatic force varies strongly with the distance [17]. Since in most cases the KPFM feedback circuit time constant is much larger than the period of the first resonance oscillations, the force minimization condition must be applied to the average force. This leads to the following relation between the measured potential and the sample potential: 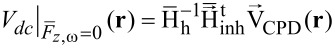, where 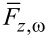 is the averaged force. In addition, 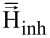 and 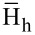 represent, respectively, the time averaged of 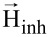 and H_h_, which are defined in Equation 1 for a certain time, i.e., for a given probe height; the product 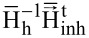 is the PSF of the system. The time averaged force was calculated by sampling the sinusoidal movement at K time points uniformly covering an oscillation period *T*_0_, so that *t*_k_ = (*T*_0_/K)·k (where k is an integer between zero and K) and the probe–sample distance is *d*(*t*_k_) = *A* sin(2π*t*_k_/*T*_0_)+*A*_0_, where *A* is the oscillation amplitude and *A*_0_ is the average height. The charge density on the probe was calculated for each probe–sample distance independently.

The magnitude of the cantilever effect on the measured potential can be explained as follows. Since the cantilever is located more than 10 μm above the sample surface, and its total lateral displacement during a high resolution scan is about 0.2 μm, its maximal angular movement relative to an axis perpendicular to the surface is on the order of 1°. Due to their large separation, the potential due to the surface dipole layer at the cantilever location can be expanded using the spherical harmonics series [18] (multipole expansion). As the angular span of the cantilever is very small during the scan, only terms with high multipole orders, (tens and higher), produce discernable angular variations. However, each multipole term decays as 1/*r*^n^ where *r* is the cantilever distance from the multipole origin, and n is the multipole order. Thus, these higher order terms in the multipole series are negligible at the cantilever location, since they decay as the reciprocal of the corresponding high power of the cantilever–sample distance. Therefore, we assumed, to a very good approximation, that the cantilever senses a constant potential during the entire scan.

To emphasize the cantilever role, we calculated separately the cantilever and tip contributions to the total vertical electrostatic force. The average force of a given geometrical model x (tip or cantilever) can be expressed using the calculated expected potential: 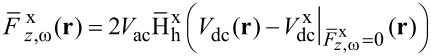; where 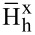 is the averaged homogeneous force coefficient and 
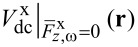
 is the nullifying force potential of the specific model x. Neglecting the mutual electrostatic interaction between the cantilever and the tip, the total force on the probe is 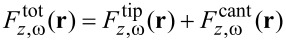. Based on the conclusion from the previous paragraph, we approximated 
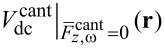
, which is the potential after nullifying only the cantilever force, by a constant. Then, by minimizing the total force we obtained:

[2]
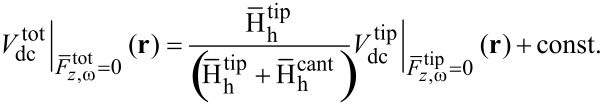


Equation 2 shows that the constant force of the cantilever introduces a factor of 
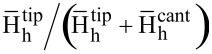
 relative to a model that takes into account only the tip. In addition, since only a scaling factor is introduced in Equation 2, the cantilever does not affect the lateral resolution, but may strongly affect the CPD absolute value, even in high resolution UHV KPFM measurements, as we demonstrate below. It should be noted that our model does not include signal-to-noise considerations, which may reduce the lateral resolution due to the above scaling.

## Results and Discussion

### Cantilever influence on the system PSF and force analysis

**Figure 1:**
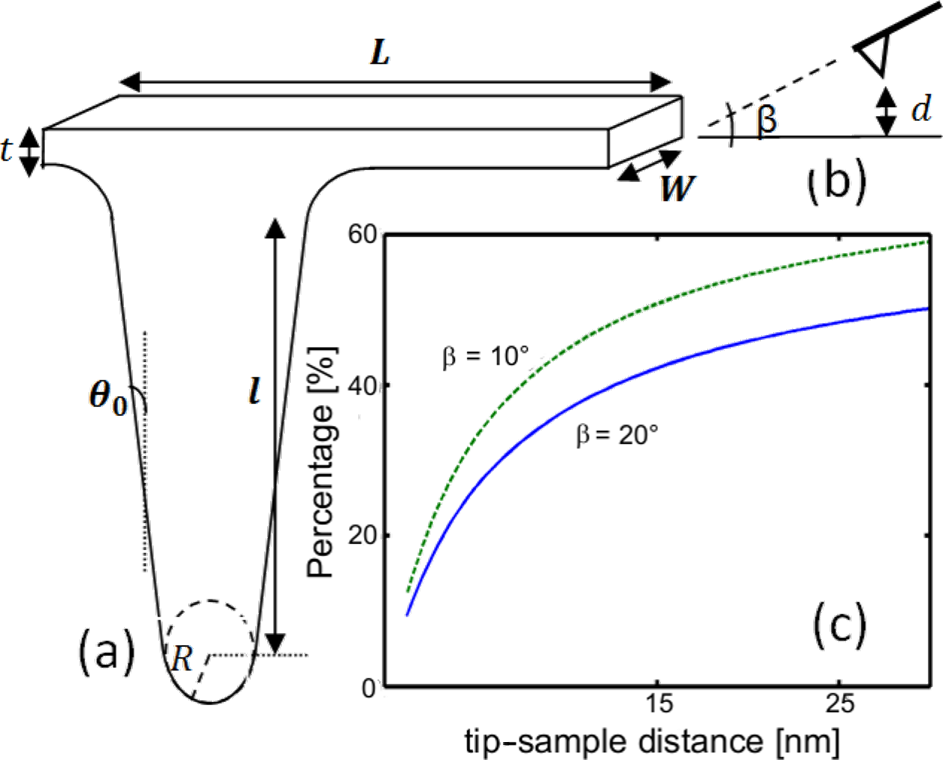
(a) Geometrical model of a tip, with cone length *l*, half-aperture angle *θ*_0_, spherical apex radius *R*, and cantilever width, length and thickness *W, L* and *t*, respectively. (b) Probe–sample cross section for a probe distance *d* from the surface, tilted at an angle β. (c) Cantilever homogeneous force contribution relative to the total homogeneous force, as a function of the probe–sample distance for two tilt angles: β = 20° (solid line) and β = 10° (dashed line), with cantilever width of *W* = 40 μm. These and all the following results were calculated for the parameter values: *R* = 30 nm, *θ*_0_ = 17.5°, *l* = 14 µm, *L* = 225 µm and *t* = 7 μm.

The influence of the cantilever was calculated for two different geometries: One comprising only a tip normal to the sample surface composed of a sphere under a cone enclosed with a spherical cap, and the other containing the entire cantilever tilted relative to the surface. The first shape does not include a tilt since it is a reference model describing a widely used geometry [3–4]. Figure 1a and Figure 1b illustrate the used variables as well as the connection between cantilever and tip cone which has a rounded shape to avoid an infinite charge density distribution on sharp edges. Figure 1c shows the calculated cantilever contribution to the total homogeneous force on the probe as a function of the probe–sample distance for two different tilt angles: β = 20° (solid line) and β = 10° (dashed line). For a probe–sample distance of 30 nm, which is frequently used in ambient KPFM, and β = 10°, the cantilever contributes around 60% of the total homogeneous force. It was observed that the cantilever influence increases with the probe–sample distance, or for smaller tilt angles, as expected.

**Figure 2:**
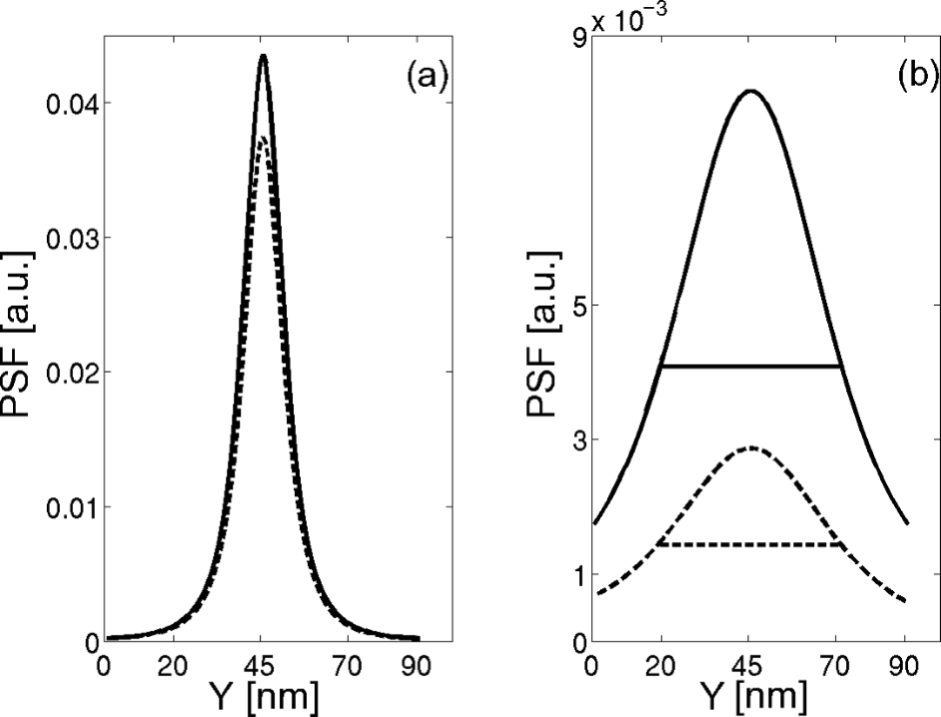
One dimensional PSF calculated for two different probe–sample distances with and without the cantilever, represented by the dashed and solid lines respectively. The model with the tip only uses β = 0° (normal to the surface) while the other one uses β = 10°. (a) Tip–sample distance of 1.2 nm, (b) tip–sample distance of 17.8 nm. The horizontal lines in (b) represent the FWHM for a probe–sample distance of 17.8 nm. The simulations were performed using *W* = 40 μm.

The effect of the cantilever on the PSF is demonstrated in Figure 2 for two different probe–sample distances with and without the cantilever, represented by the dashed and solid lines, respectively. For a probe–sample distance of 1.2 nm (Figure 2a), the maximum value of the cantilever PSF decreased by about 85% compared to the tip PSF. At a probe–sample distance of 17.8 nm, the presence of the cantilever reduced the PSF peak by almost a factor of 3 compared to the PSF computed without the cantilever. The horizontal lines represent the full width at half maximum (FWHM) for the two cases and demonstrate the conclusion that the cantilever hardly affects the measurement resolution. It should be emphasized that the difference between the two cases stems not only from the cantilever, but also from the tilt of the probe relative to the surface.

**Figure 3:**
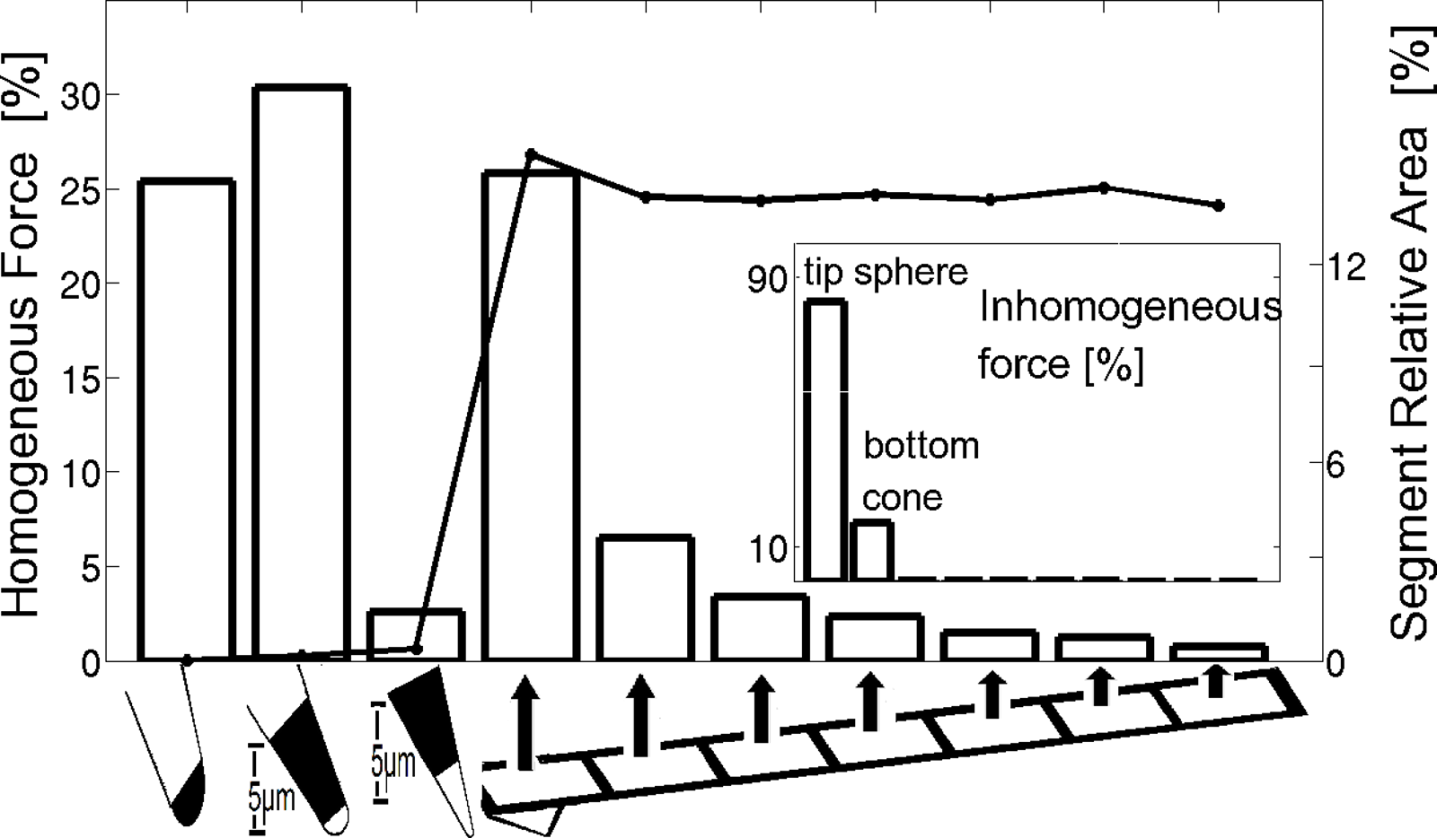
Left axis: Relative magnitude of the homogeneous force distribution on different fractions of the probe; right axis: The relative area percentage of each of the segments. The graph was calculated for β = 20° with a probe–sample distance of *d* = 17.8 nm. The probe was divided into ten segments (presented from left to right) – the bottom sphere, the bottom part of the cone (vertical length of 5 µm), the top part of the cone (vertical length of 5 µm) and seven segments of the cantilever each with an horizontal length of 26.7 µm (the outer most segment of the cantilever does not include any part of the cone). The inset figure represents the relative inhomogeneous force for each segment as a percentage of the total inhomogeneous force on the probe.

Figure 3 displays the relative homogeneous force contribution of the various parts of the probe normalized to the total homogeneous force (left axis), for a probe located 17.8 nm above the surface. Each bar corresponds to a different part of the probe defined as follows (from left to right): The bottom sphere of the tip, the bottom and top parts of the cone (each having a vertical length of 5 μm), and seven segments of the cantilever each with an equal length of 26.7 μm, with the first segment located closest to the tip. The spherical tip apex and the bottom part of the cone contribute 25% and 30% to the overall homogeneous force, respectively. The rest of the force stems mostly from the cantilever, especially from the two segments which are nearest to the tip which contribute 25.8% and 6.5% each. The effect of the cantilever segments decreases the further away each segment is from the tip. This is due to the tilt of the cantilever which increases the distance of each segment from the sample surface as we move along the cantilever away from the tip. Nevertheless, since the cantilever area is very large even the remaining five outermost segments contribute about 9.2% of the total force. The right axis in Figure 3 presents the relative area of each part of the probe out of the total probe surface area. The area of the first two parts is significantly smaller than that of the cantilever. In addition, though the upper part of the cone has a much larger surface area than the lower one, it has a very small effect on the overall force, since its surface area is not large enough to compensate for the decay in the force – which is a result of the increasing distance from the sample.

The inset of Figure 3 shows the relative inhomogeneous force magnitude distribution along the probe using the same segments. The force was calculated for a square sample (192 nm by 192 nm) having a potential difference of 1V relative to an infinite earthed substrate around it. It was observed that the spherical apex of the tip and the bottom part of the cone contribute 82.7% and 17.2%, respectively, of the inhomogeneous force, while the contribution of the rest of the probe was negligible. This demonstrates the profound effect of the tip apex on the KPFM resolution and, consequently, the minor influence of the cantilever.

Further calculations showed that at smaller probe sample distances the homogenous force contribution of the tip apex is higher. At a probe–sample distance of 1.2 nm (a typical distance in ultra-high vacuum measurements) the tip apex contributes 83% to the homogenous force, the cone lower segment contributes 7.3%, and the entire cantilever contributes only 8.4%.

### Comparison with experimental results

**Figure 4:**
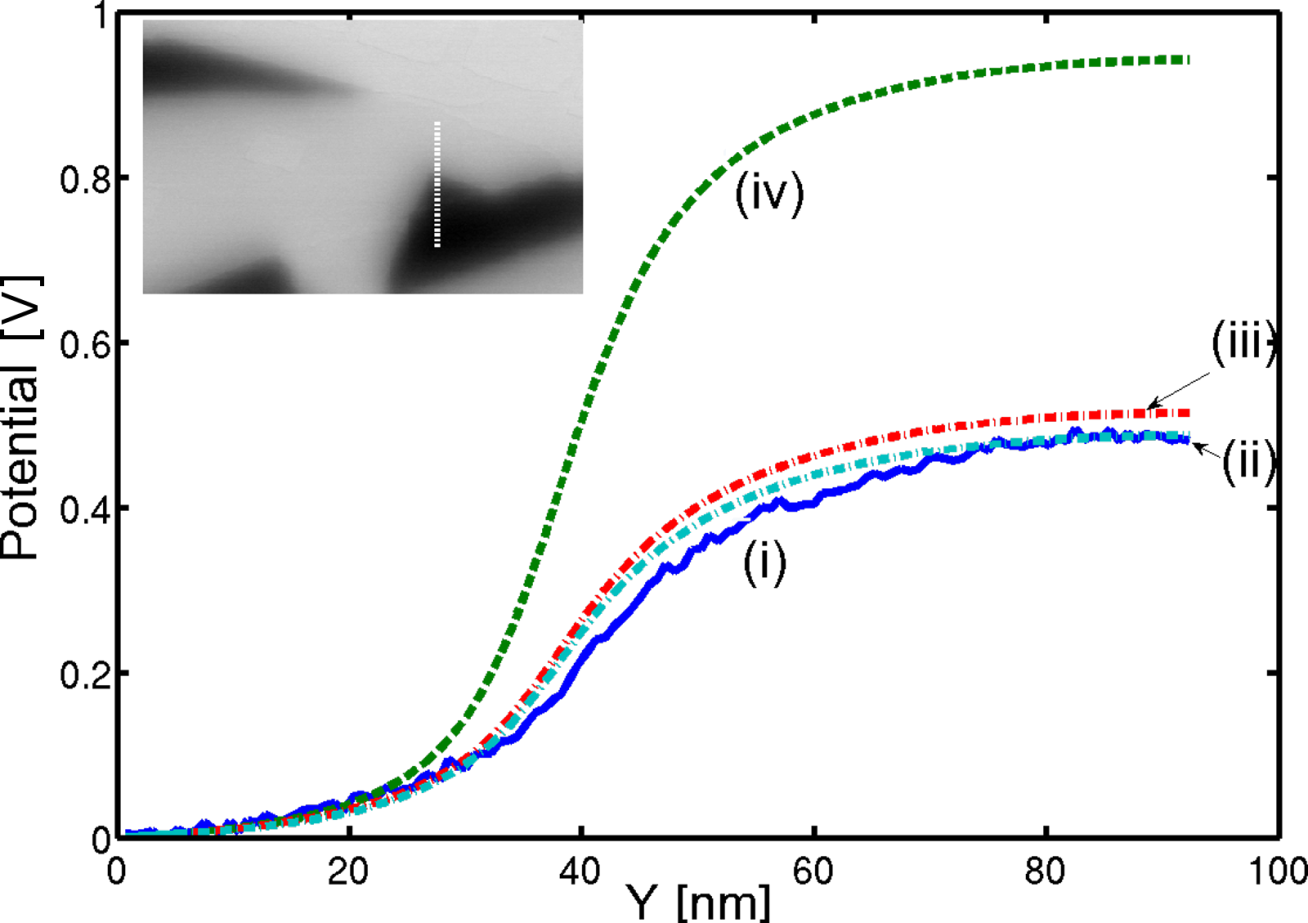
Line section (vertical line at inset figure) for KPFM simulation with different cantilever geometries. (i) Original measurements, (ii) *W* = 40 µm, β = 10° (iii) *W* = 40 µm, β = 20°, (iv) probe without cantilever with β = 0° (normal to the surface). Inset figure: Single pass AM-KPFM measurements of NaCl thin films grown on Cu(111) [19] with a cantilever first resonance amplitude of 20 nm and with a minimum distance of 1 nm. The dashed line represents the line section of the simulations.

The above analysis was applied to high resolution UHV KPFM measurements of NaCl thin films grown on Cu(111) [19]. The simulation was performed by convolving the two-dimensional PSF with the theoretical surface potential difference between Cu and NaCl, where we assumed that the actual CPD landscape is approximately identical to the measured topography. Therefore, we used the measured topography as a rough estimate for the theoretical surface potential. Figure 4 shows a comparison between the measured CPD curve (i) and the simulated potential along a single line section (dashed line in the inset image). Curves (ii) and (iii) were calculated for a probe that includes a cantilever with two different tilt angles, and curve (iv) corresponds to a vertical tip. The calculation that included the cantilever shows a good agreement with the measurements both in terms of the resolution and the absolute CPD value. Comparison of curve (ii) to curve (iii), which represent tilt angles of 10° and 20°, respectively, shows that the exact angle has a weak effect. The model that includes only the tip shows a good agreement in terms of spatial resolution, but is about a factor of 2 larger than the absolute CPD value. Additional simulations show that changing the cantilever width may have a large effect on the results.

**Figure 5:**
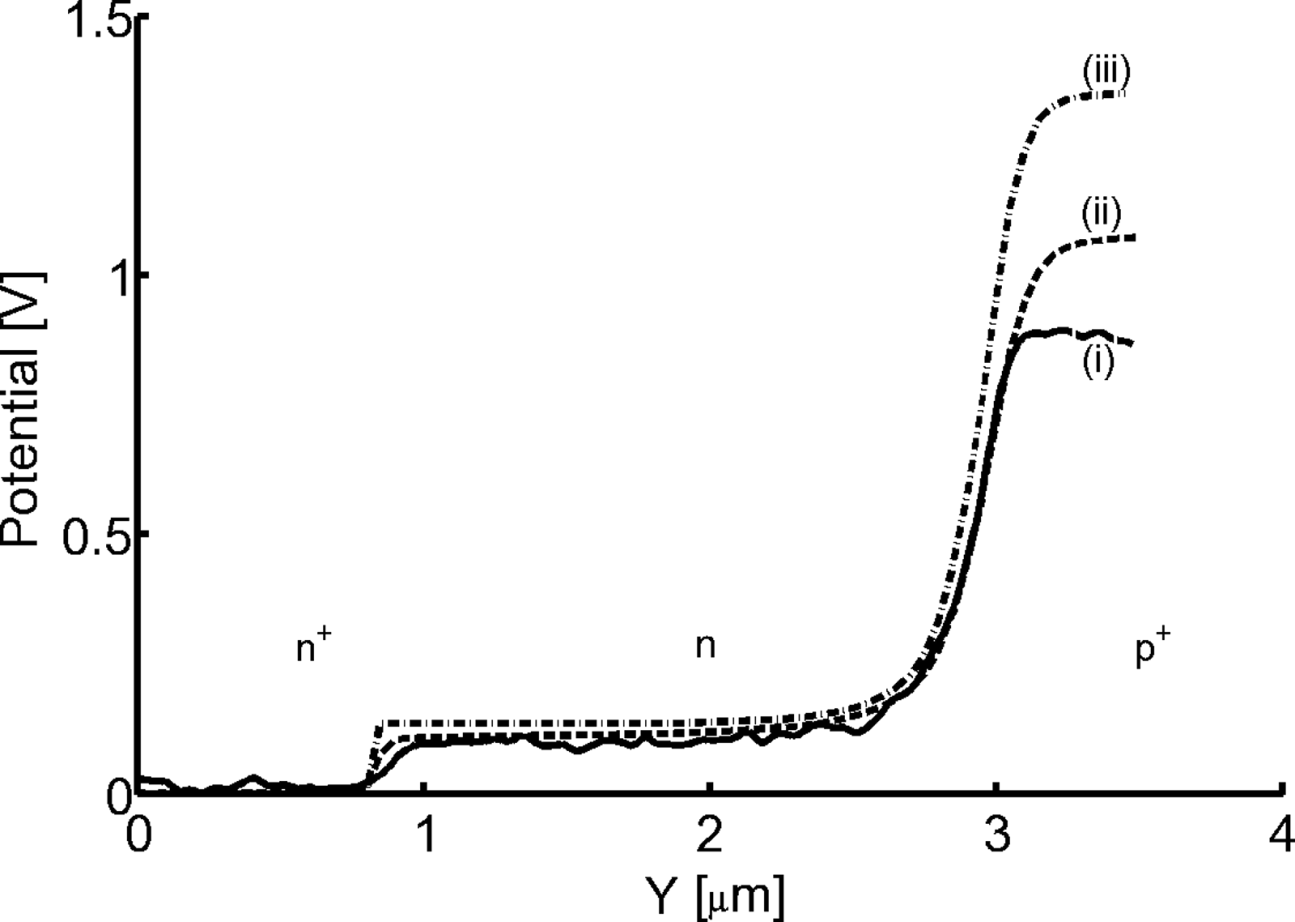
Line section of UHV KPFM (i) measurements [20], (ii) simulated, and (iii) theoretical potential distribution of InP(100) p^+^nn^+^ junction. The measurements were performed at single pass with cantilever first resonance amplitude of 3 nm with a minimum distance of 0.5 nm. The simulation was performed using the following probe geometry: *R* = 30 nm*,* β = 10°.

We also demonstrate the effect of the cantilever on UHV KPFM measurements of a cleaved InP(100) p^+^nn^+^ junction [20]. As observed in Figure 5, the measured potential difference across the p^+^n part of the junction (i) is ~0.9 V, which is smaller than the theoretical difference of around 1.35 V (iii) (calculated assuming an absence of surface states). Curve (ii) is the potential profile obtained by convoluting the theoretical junction potential (iii) with the PSF of the specific probe used in the experiments. It was observed that even far from the junction, i.e., deep inside the p^+^ InP, the cantilever induced a potential offset of about 22% relative to the theoretical profile. This is in agreement with our analysis that the cantilever has a large influence on the absolute CPD value even above a relatively large equipotential area. The difference of ~0.15 V between the measurement and the convoluted profile may be attributed either to surface states or to a slightly different cantilever geometry.

### The role of the cantilever oscillations

The analysis in the previous sections assumed that the cantilever shape is rigid during the measurement. In practice, the cantilever bends according to its mechanical properties. This has two implications on the force analysis presented above: The first is related to a different probe–sample distance profile which stems from the cantilever first resonance shape, while the second is a result of the change in the cantilever shape in its second resonance mode; this leads to a differentially weighted effect of the electrostatic forces along the cantilever. These two effects were analyzed and are discussed below.

#### The effect of the first resonance

In either the single or dual pass KPFM methods the cantilever oscillates at the frequency of its first resonance in order to measure the surface topography in the non-contact mode. In the previous sections the cantilever was considered rigid, meaning that during the calculation of 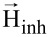 and H_h_ of Equation 1 along the vertical tip movement, only the minimal probe–sample distance changed while the cantilever geometry was considered constant. In practice the cantilever beam oscillates according to the boundary conditions of a clamp-free beam. The cantilever was modeled as a rectangular prismatic beam with one end restrained and the other one free. We used the beam fundamental mode formula, while neglecting deformations that may be introduced by the presence of the tip load at the end of the cantilever or by additional forces between the sample and the tip. This was done in order to evaluate the main influence of the oscillation without adding unnecessary complexity. Assuming that the cantilever is clamped at *y*' = 0, the vertical deformation along the *y* axis, and as a function of time, is given by [21]:

[3]
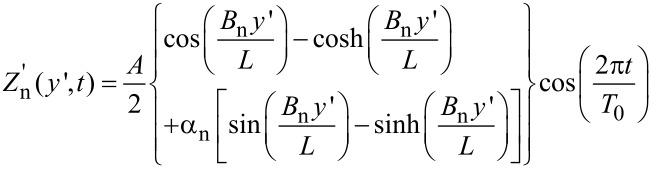


where for the first mode (n = 1) *B*_1_ = 1.975, α_1_ = −0.731, *L* is the cantilever length and, as before, *T*_0_ is the oscillation period and *A* is the oscillation amplitude. The *y*' and *z*' axes are rotated by β degrees relative to the main coordinates (*y*, *z*) (see also Figure 7).

As before, the oscillatory movement was uniformly sampled; for each discrete time sample an entirely new geometry was established according to the deformation of Equation 3 and the average probe–sample height, *A*_0_. For each configuration, the tip was positioned perpendicular to the free edge of the cantilever and the clamped edge of the cantilever was always at the same position. All these geometries were created using Patran's® command language (PCL) used to create automatically the entire geometry and mesh at any given time.

**Figure 6:**
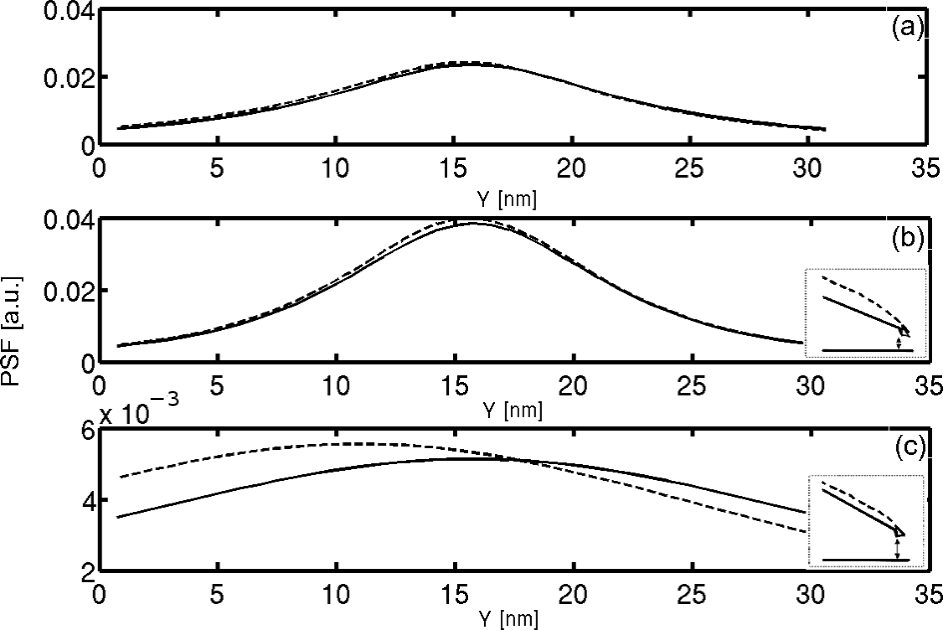
Beam deflection influence on PSF. The dashed line represents the PSF of a deflected beam while the solid one is for a stiff cantilever. Simulation was performed with cantilever first resonance amplitude of 20 nm with a minimum distance of 1 nm. (a) PSF comparison after averaging; (b) comparison for a probe located at a distance of 1.2 nm above the surface; (c) comparison for a probe located at a distance of 11.9 nm above the surface. Inset figures at (b) and (c) illustrate the deflected and stiff beams that were used for the calculations.

The influence of the beam deflection is shown in Figure 6. In all the three plots, the cantilever PSF (dashed lines) is compared to the rigid cantilever (solid lines); both were calculated for a cantilever oscillating with an amplitude of 20 nm and a minimum probe–sample distance of 1 nm. Figure 6b and Figure 6c present a comparison for a probe positioned at distances of 1.2 nm and 11.9 nm, respectively, above the surface. The inset figures illustrate the shape and position of rigid (solid line) and deformed (dashed line) cantilevers, emphasizing that the comparison is performed while maintaining the same minimum probe–sample distance in both cases. Figure 6b shows that at the lowest point of the oscillation there is a weak influence of the cantilever deformation on the PSF. Close to the equilibrium point, shown in Figure 6c, the significant difference between the two PSFs is that they are shifted, which clearly visible by comparing the peak positions. This offset of about 5 nm results from the change in the cantilever shape which changes the tip angle. The averaged PSF, which is the average over the entire oscillation cycle, is presented for both cases in Figure 6a. It can be seen that the overall impact of the cantilever deformation, both on the average PSF and at any given probe–sample distance, is negligible. Therefore, we conclude that a simple model of a rigid cantilever is an adequate approximation.

#### The effect of the second resonance

**Figure 7:**
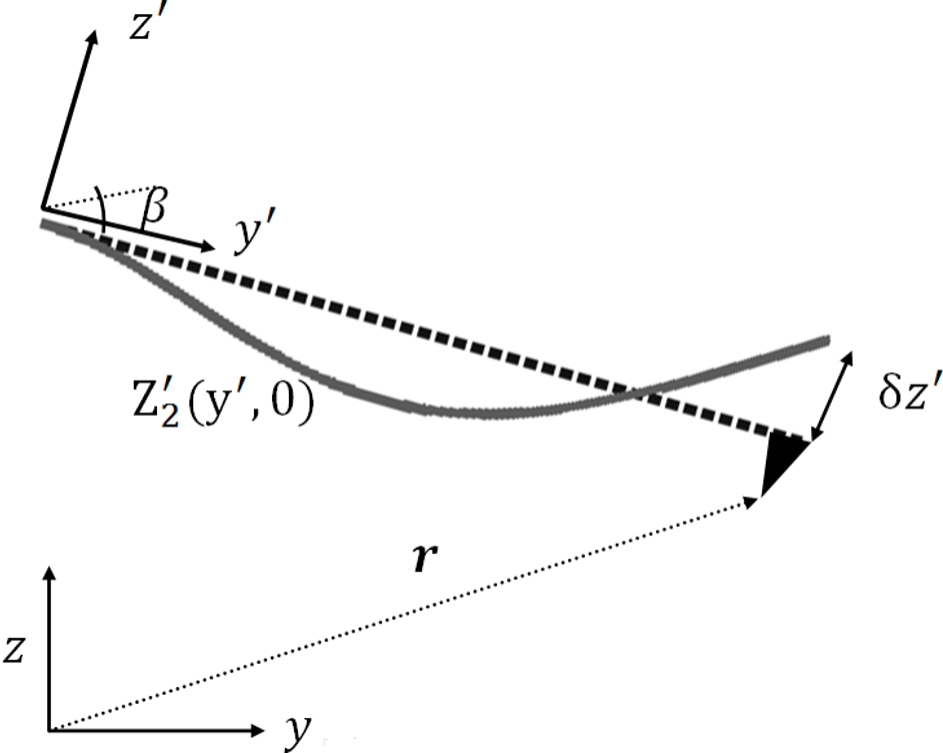
Second harmonic deflection relative to the cantilever at its rest position. The free edge deflection of the cantilever is δ*z*'. All the other amplitude values along the cantilever are calculated relative to this deflection.

In most AM-KPFM single pass measurements an external AC bias, at a frequency ω of the second resonance of the beam, is applied to the entire probe. This oscillation, shown in Figure 7, is minimized by applying an additional DC bias to give the CPD. In the previous sections this was modeled by nullifying the entire electrostatic force acting on the probe. However, this analysis is not accurate since the electrostatic forces at different points along the cantilever have a different effect on the beam edge amplitude. As an example, consider a point along the cantilever which has zero amplitude (e.g., the end point which is held mechanically fixed). The forces acting at this point do not affect the amplitude measured by the detector, and therefore should not be considered in the electrostatic force minimization.

We first assumed that at a frequency ω (the second mechanical resonance) the beam is always deformed according to its second harmonic movement. We can assume that this is the only relevant mode, since it is the only frequency passed by the filter before the KPFM feedback circuit. Assuming, once again, that the beam deflection is purely harmonic, its deflection is given by Equation 3 with the coefficients B_2_ = 4.69, α_2_ = −1.018, corresponding to the second mode (n = 2). In order to analyze the influence of the second resonance, we use the concept of virtual displacement [22] which states that the system equilibrium is obtained when the total external (virtual) work acting on the beam is zero. For a given time *t* = 0, assuming that the free edge of the cantilever (*y*' = *L*) undergoes a small (virtual) displacement δ*z*', we can determine the relative displacements of every point along the cantilever by using *A* = δ*z*' in Equation 3. In this situation, the entire virtual work *W*_z_(**r**) done by the external electrostatic forces in the *z* direction, for a probe positioned at **r**, is given by

[4]
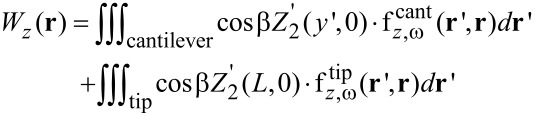


where 
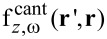
 and 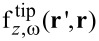 are the local forces acting at point **r**' on the cantilever and the tip, respectively, when the probe is located at **r**. In addition, **r**' corresponds to the rotated coordinate system (*x*', *y*', *z*'). Since the tip is located at the end of the beam, it experiences a constant amplitude. Figure 7 illustrates the second harmonic bending described by the function *Z*'_2_(*y*', 0) with an edge amplitude of *A =* δ*z*' relative to a probe tilted at an angle β and positioned at **r** in the main fixed coordinate system.

The steady state is reached when *W*_z_(**r**) is minimized instead of the total electrostatic force. This is achieved by multiplying the force over each boundary element by its relative virtual displacement. We define a diagonal matrix **Z** whose diagonal elements are equal to the relative displacement for each cantilever element and equal to 1 for each tip element. The overall virtual work function may then be written as

[5]
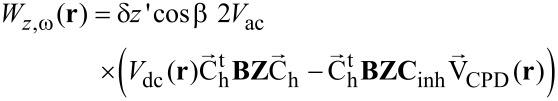


By nullifying the above expression we may achieve the new PSF of the system, similar to the process described in the Experimental section.

**Figure 8:**
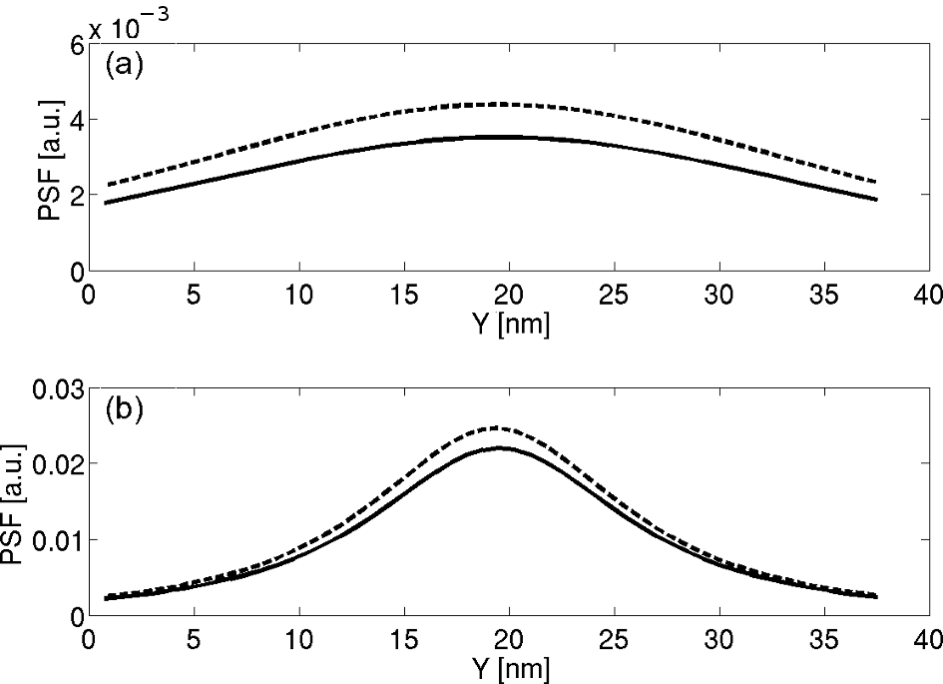
Second harmonic weighting influence on the PSF. Dashed lines: PSF calculated with the second harmonic displacement, using virtual work equilibrium. Solid lines: PSF calculated for a rigid cantilever. (a) Probe–sample distance *d* = 11.4 nm; (b) averaged PSF for amplitude of 20 nm with a minimum distance of 1 nm. For both cases the cantilever parameters were *W* = 40 μm, β = 20°.

Figure 8 shows the effect of the second harmonic oscillations on the calculated PSF, for a probe–sample distance of 11.4 nm (a) and for an average PSF calculated for a first resonance movement with a minimal probe–sample distance of 1 nm and amplitude of 20 nm (b). The figure shows that the introduction of the second harmonic weighting has changed only the PSF height and not its shape, since it influences only the cantilever. In addition, it caused the PSF to increase by around 20% and 10% for a probe–sample distance of 11.4 nm and for the averaged PSF, respectively. The impact of the second harmonic oscillations is limited, since as shown in Figure 3, the dominant contribution of the cantilever to the homogeneous force stems from the areas closest to the tip. These areas resonate with similar amplitudes to that of the tip and therefore their relative displacement will be close to one. This additional refinement of the model does not have an entirely negligible influence on the PSF. However, since most of the impact of the cantilever remains the same, as in the model with a rigid cantilever, using such a model may provide a sufficiently accurate approximation. It should be noted that the above analysis will be different in the dual pass technique, since the applied bias frequency usually differs from the second resonance of the beam.

## Conclusion

We have used the BEM method to calculate the cantilever contribution in KPFM measurements. By analyzing the force expression, we showed that although the cantilever may have little effect on the measurement resolution, it has a profound influence on the absolute CPD value. The influence of the cantilever has a direct relation to the probe–sample distance and an inverse relation to the probe–surface angle. It was found that even at probe–sample distances in the range of several nanometers, the absolute CPD may change by as much as 50% if the cantilever contribution is neglected. We have applied our analysis to UHV KPFM measurements and obtained good agreement both in the resolution and in the absolute value of the measured potential. This suggests that the cantilever must be taken into account in quantitative surface potential measurements. Longer tips or FM-KPFM will reduce the cantilever contribution and improve the measurement precision.

In the second part of this paper, we calculated the influence of the cantilever deformations on the measured KPFM. It was found that the exact cantilever shape in its first resonance has a very small effect, while the second resonance deformation has a larger effect on the PSF and thus should be considered where high surface potential accuracy is required.

## Appendix – full matrix definitions

In this appendix we explicitly define the matrices that are used in the paper.

We define 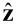 as the unit vector pointing in the *z* direction and **r**_i_ as the location of the center of the *i*th boundary element of the probe's surface. Each probe element is assumed to have a constant surface charge density. The *ij*th element of matrix **G** is given by

[6]
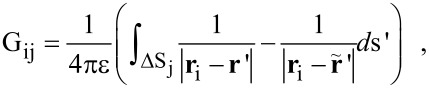


where 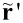 is the location of the image charge of the probe's *j*th element relative to an infinite earthed plane, so that if **r**' = (*x*', *y*', *z*') then 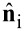 = (*x*', *y*', –*z*'). The integral is performed over the *j*th surface element of the probe. The diagonal of matrix **B** is defined as

[7]
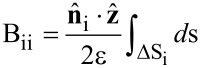


where 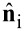 is the outward normal unit vector to the *i*th surface element. The integral is performed over the probe's *i*th surface element.

The sample surface potential is discretized using uniform square elements according to the resolution of the scan, denoted Δ. The center of the *k*th surface element is located at **r**_k_ = (k_x_Δ, k_y_Δ) where both k_x_ and k_y_ are integers. The *ik*th element of matrix **D** is described as

[8]
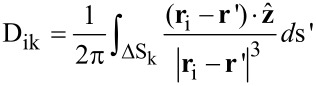


where the integral is performed over the *k*th element of the sample surface.

The *k*th element of the vector 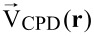 is obtained as

[9]
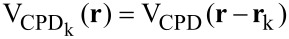


where V_CPD_(**r**) is the continuous CPD function of the sample and **r** represents the lateral position of the probe.

The measured potential over the probe for each location **r**, *V*_p_(**r**), is a superposition of the potential induced by the charge distribution over the probe and the potential induced by the sample:

[10]
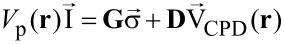


where 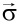 is a vector representing the charge distribution on each boundary element of the probe and 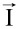 is a vector whose elements are equal to one. The probe's charge density is extracted using this equation. By inserting the charge density into the Maxwell stress tensor, we obtain Equation 1.
